# Dexmedetomidine alleviates acute kidney injury in a rat model of veno‐arterial extracorporeal membrane oxygenation

**DOI:** 10.1186/s40635-025-00720-4

**Published:** 2025-01-30

**Authors:** Min Yu, Shilin Wei, Xueyang Shen, Junjie Ying, Dezhi Mu, Xiangyang Wu, Yongnan Li

**Affiliations:** 1 Department of Anesthesiology, Lanzhou University Second Hospital, Lanzhou University, Lanzhou, China; 2 Department of Thoracic Surgery, Lanzhou University Second Hospital, Lanzhou University, Lanzhou, China; 3 Department of Neurology, Lanzhou University Second Hospital, Lanzhou University, Lanzhou, China; 4 Department of Pediatrics, Key Laboratory of Birth Defects and Related Diseases of Women and Children, Ministry of Education, West China Second University Hospital, Sichuan University, Chengdu, 610041 China; 5 Department of Cardiac Surgery, Lanzhou University Second Hospital, Lanzhou University, No. 80, Cuiyingmen, Chengguan District, Lanzhou, 730030 China

**Keywords:** Acute kidney injury, Veno‐arterial extracorporeal membrane oxygenation, Dexmedetomidine

## Abstract

**Background:**

Although extracorporeal membrane oxygenation (ECMO) is an effective technique for life support, the incidence of acute kidney injury (AKI) during ECMO support remains high. Dexmedetomidine (DEX), which has been widely used for sedation during ECMO, possesses several properties that help reduce the occurrence of AKI. This study aimed to investigate the protective effect of DEX on kidney function during ECMO.

**Methods:**

A total of 18 male Sprague–Dawley (SD) rats were randomly divided into three groups: Sham, ECMO, and ECMO + DEX groups. ECMO was established through the right jugular vein for venous drainage and right femoral artery for arterial infusion and lasts for four hours. Hematoxylin and eosin staining was used to evaluate the kidney Paller score for the rats in each group. Enzyme-linked immunosorbent assay was used to measure the levels of kidney injury biomarkers and cytokines in the serum. Reagent kits were used to measure the blood urea nitrogen (BUN) and creatinine (Cr) levels, which helped determine kidney function. Immunohistochemical staining was used to evaluate neutrophil infiltration in the kidney.

**Results:**

The pathological Paller score was substantially lower in the ECMO + DEX group. The levels of Kidney Injury Molecule-1 (KIM-1) and N-acetyl-β-D-glucosaminidase (NAG) were also significantly reduced. The kidney functionality, as indicated by BUN and Cr, was significantly improved compared with the ECMO group. The levels of cytokines IL-6, IL-1β, and TNF-α, were also significantly decreased in the ECMO + DEX group.

**Conclusion:**

This study demonstrated that dexmedetomidine could reduce inflammatory response and alleviate AKI during ECMO support.

## Introduction

For patients with multiple organ failure, especially heart and/or respiratory system failure or dysfunction, extracorporeal membrane oxygenation (ECMO) is an effective life-saving technique that has been widely used since the 1970s [1, 2]. ECMO support technique can be divided into two major types based on the blood vessel used for perfusion: veno-venous (VV) and veno-arterial (VA). Although ECMO is an optimal solution for patients with life-threatening diseases who are unresponsive to conventional treatment, the complications associated with ECMO can be catastrophic for critically ill patients. The ECMO-related complications are broadly divided into two basic types: ECMO equipment-related complications (circuit or cannula events and pump malfunction), and physiological complications (hemolysis, stroke, bleeding, infection, limb ischemia, and severe organ damage). Acute kidney injury (AKI) is the most commonly reported ECMO-related complication, which has a poor prognosis and high mortality rate [3–5]. Chaves et al. reported that the incidence of AKI ranged between 70 and 85% for patients on ECMO support. More importantly, for patients with AKI, the ECMO technique is associated with a higher mortality rate of ~ 80% [6]. An animal study by Antal Szabó-Biczók et al. found significantly decreased renal function with signs of structural damage in pigs with ECMO [7]. Therefore, to improve the outcomes of these critically ill patients on ECMO support, it is crucial to reduce the incidence of ECMO-related AKI.

Sedation and analgesia are principal components for better management of patients on ECMO. To achieve better blood flow in the circuit, optimize ventilation, and minimize oxygen consumption, the Extracorporeal Life Support Organization (ELSO) recommends sedation to mild anesthesia during ECMO support for the first 12 to 24 h [8]. However, the optimal pharmacological management of sedation and analgesia has not been defined clearly in patients on ECMO support.

Dexmedetomidine (DEX), a selective α2-adrenoreceptor agonist with sedative, analgesic, and anti-anxiety effects, has been used in critically ill patients, including those on ECMO therapy [9]. DEX is also beneficial for treating AKI owing to its anti-sympatholytic characteristics and highly selective α2 adrenergic receptor agonist effect [9]. The α2-adrenoreceptors can be found widely distributed in the peritubular vasculature and the distal and proximal tubules of the kidney [10]. Several studies have demonstrated that DEX can decrease the endotoxin-induced upregulation of inflammatory molecules and attenuate renal function deterioration associated with AKI [11, 12]. However, it remains unclear whether DEX can reduce the incidence of AKI in patients who are on ECMO support. Therefore, the purpose of this study was to explore whether DEX can alleviate AKI in a rat model of VA-ECMO.

## Methods

### Animal preparation

This study was approved by the Lanzhou Second Hospital, Lanzhou University, Gansu. All animals received humane care in compliance with the “Principles of laboratory animal care” formulated by the National Society for Medical Research and the “Guide for the care and use of laboratory animal resources” published by the US National Institute of Health (NIH publication No. 85-23, revised 1996). All rats used in the study were purchased from the Veterinary Institute, Chinese Academy of Agricultural Sciences, Lanzhou, China. A total of 18 male Sprague–Dawley (SD) rats weighing 350 ± 50 g were randomly divided into three groups: sham, ECMO, and ECMO + DEX groups. The experimental animals were numbered successively, starting from 1 to 18; six numbers were randomly selected first as the Sham group, then another six numbers were randomly selected as the ECMO group, and the remaining six numbers were selected as the ECMO + DEX group.

### Procedures and drug administration

**Fig. 1:**
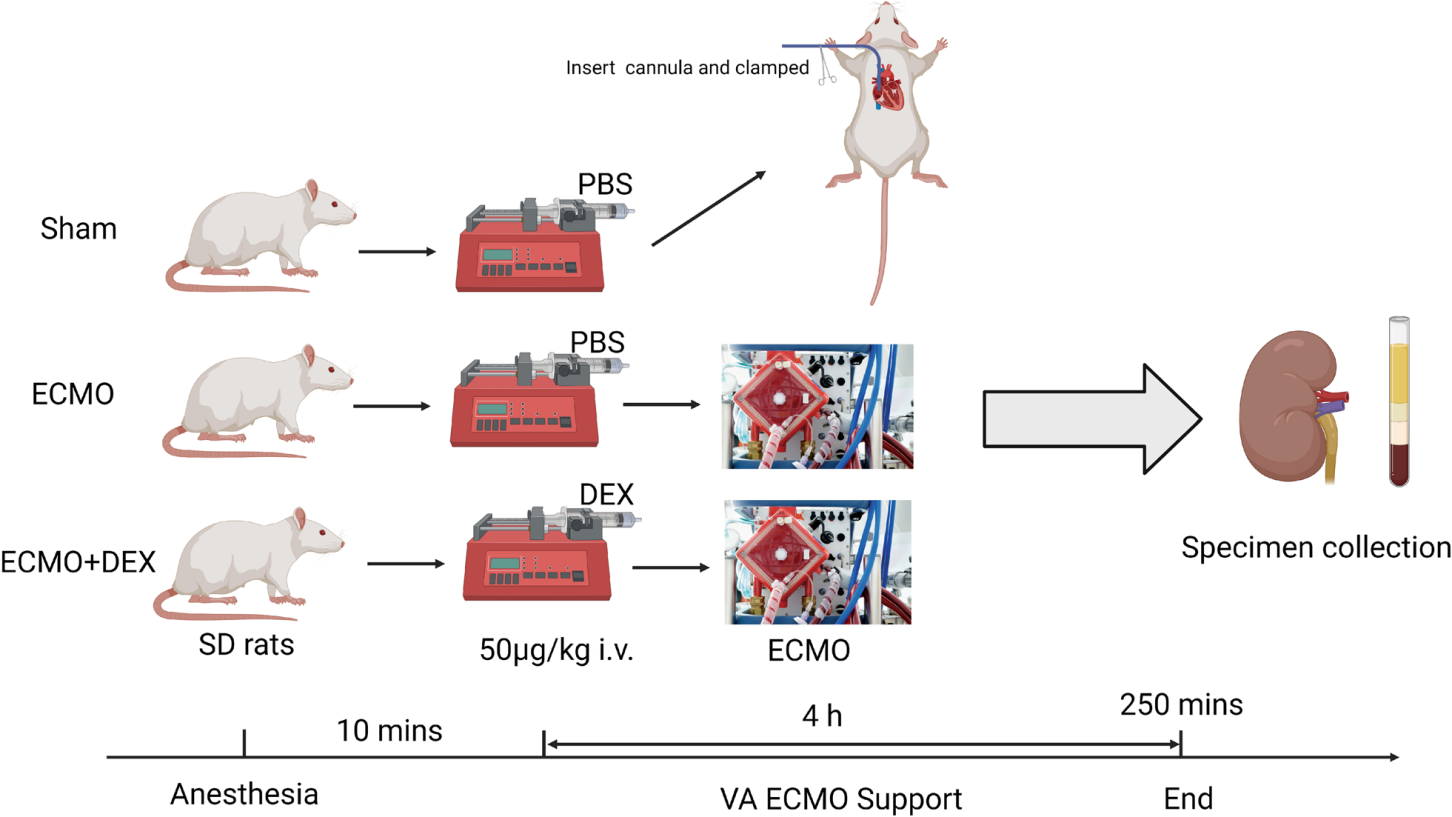
Flow diagram of the experimental grouping. Created using Biorender

Rats were anesthetized using 5% sevoflurane in oxygen for 3 to 5 min, in a plexiglass chamber. Volume-controlled ventilation was provided at a respiratory rate of 70–75 breaths/min, a tidal volume of 6 ml/kg, and a positive end-expiratory pressure (PEEP) of 2 cmH_2_O. The gas flow set on the membrane was based on our previous experiment, about 30 mL/min, the blood flow of the VA-ECMO is 80–90 ml/kg/min. The heparin was administrated at 300 U/kg. At the middle of the experimental arterial blood gas was analyzed, NaHCO3 was administered based on the results to control CO2. During subsequent surgical preparation, anesthesia was maintained using 2.0–2.5% sevoflurane. Surgery was performed using an aseptic technique, and all surgical fields were subsequently infiltrated with 1% lidocaine. ECMO cannulas were inserted in rats in the Sham group as well, and after the same prime fluid (consisting of Ringer's solution (2.5 ml) and 6% HES130/0.4 (2.5 ml)) volume and heparin in the ECMO group was given, right jugular vein cannula was clamped, and right femoral artery cannula was preserved for blood pressure monitoring. The animals in the Sham group also received heparin to reduce experimental heterogeneity and prevent thrombosis. In the ECMO + DEX group, the rats were administered DEX (50 µg/Kg) before VA-ECMO support to explore the protective effect of DEX on the kidneys (Fig. 1). Dexmedetomidine was given through the right jugular vein cannula as a bolus before ECMO was initiated. The configuration of VA-ECMO in rats has been described in our previous studies [13]. Briefly, ECMO was established through the right jugular vein for venous drainage and right femoral artery for arterial infusion, and after four hours of ECMO modeling, arterial blood from the femoral artery was collected for blood gas analysis (EG7 + , iStar, Abbott Co. Ltd) then rats were euthanatized using overdose sevoflurane, serum, and kidney tissues were collected for further analysis.

### Hematoxylin–eosin staining

Kidney tissues were subject to paraffin embedding and subsequently cut into 4-μm-thick sections. Hematoxylin (#H8070, Solarbio) and Eosin (G1100, Solarbio) staining was performed on the sections as per the standard protocol, and the histological changes were observed under a light microscope. Subsequently, a pathologist determined the Paller score in a double-blind setting by assessing brush border loss, tubular epithelium smoothness, cytoplasmic vacuolization, cell necrosis, and tubular lumen obstruction [14]. The modified Paller score, which indicates the degree of kidney injury in one visual field, was calculated by summing up the scores (ranging from 0 to 8) for tubular epithelium smoothness (0,1), loss of brush border (0,1,2), cytoplasmic vacuolization (0,1), tubular lumen obstruction (0,1,2), and cell necrosis (0,1,2), observed in that field.

### Enzyme-linked immunosorbent assay

The relative levels of IL-6, IL-1β, and TNF-α in the serum and the levels of kidney injury molecule-1 (KIM-1) and N-acetyl-β-D-glucosaminidase (NAG) in the serum were determined using the respective ELISA kits (Mlbio, Shanghai, China), according to the manufacturer’s recommendations.

### BUN and Cr assay kit

The levels of BUN and Cr in the serum were detected using the biochemical reagent kits (Nanjing jiancheng Bioengineering Institute, Nanjing, China), according to the manufacturer’s recommendations.

### Immunohistochemistry

Immunohistochemical staining was performed using the mouse/rabbit streptomycin ovalbumin-biotin assay system (#SP-9000, ZSGB-BIO, China), as per the standard protocol. The primary antibody used in the assay was the rabbit anti-MPO antibody (1:1000, #ab78486, Abcam). The image was visualized using a Zeiss microscope (Zeiss, Germany) and processed using the Zeiss software.

### Statistical analysis

All statistical analyses were performed using GraphPad Prism 7.0 (GraphPad, USA), and all graphs were obtained using the same software. The data were expressed as mean ± SD. The normality of the distribution was studied using the Shapiro–Wilk test. Data from perioperative physiological parameters were analyzed using repeated measurements of analysis of variance (ANOVA). Normally distributed data from the two groups were compared using the unpaired Student’s t-test, while the non-normally distributed data were compared using the non-parametric Mann–Whitney U-test. ANOVA, followed by Bonferroni post hoc test, was used for comparisons across the three groups. A p-value < 0.05 (2-tailed) was considered statistically significant.

## Results

### Hemodynamic and blood gas characteristics

**Fig. 2:**
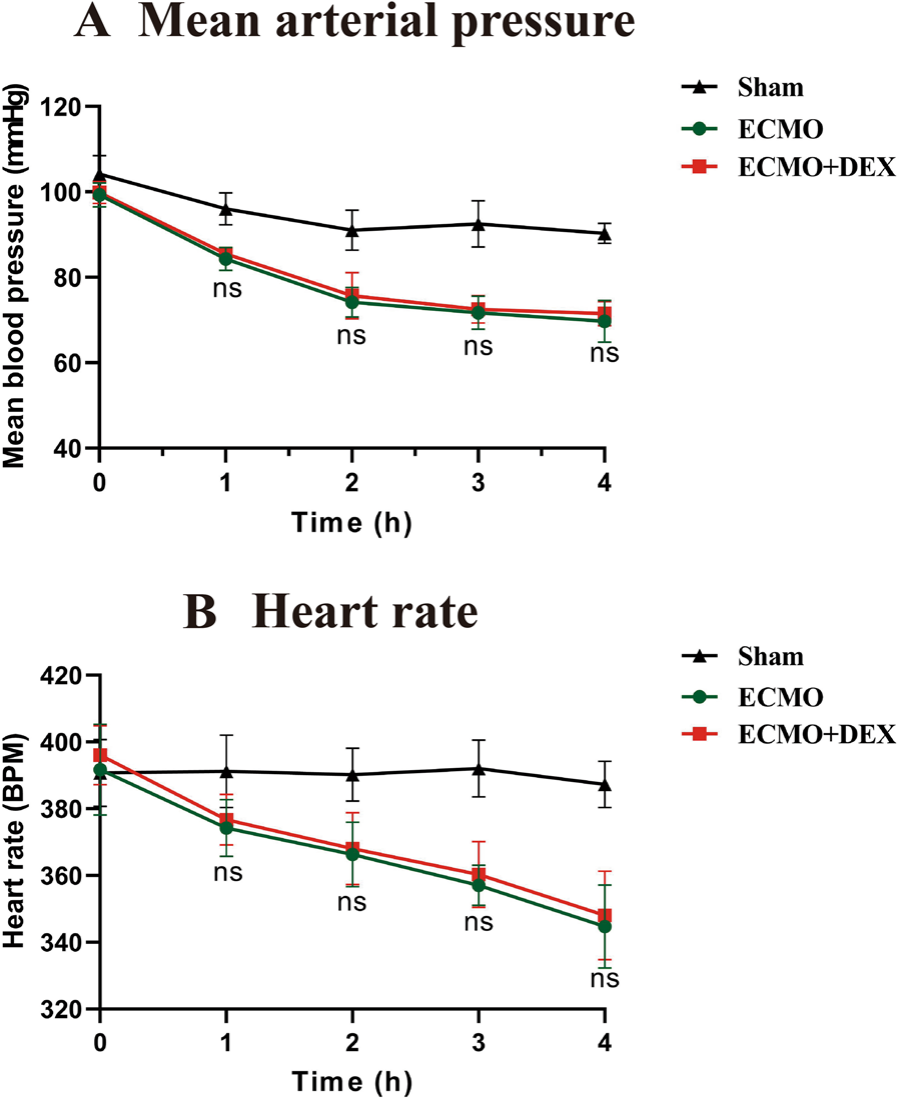
Mean arterial pressure and heart rate during the experimental in different groups

Mean arterial pressure (MAP) and heart rate at T0 (Baseline), T1 (1 h), T2 (2 h), T3 (3 h), and T4 (end of experiment) were recorded to evaluate the hemodynamic state (Fig. 2A and B). There is no significant difference between ECMO and ECMO + DEX group at T1, T2, T3, and T4. Furthermore, arterial blood was collected at the end of the modeling to assess blood gas and biochemical parameters (Table 1).

**Table 1:** Metabolic changes at the end of the modeling

Biochemical parameter	Sham	ECMO	ECMO + DEX	P value
pH	7.350 ± 0.058	7.316 ± 0.121	7.309 ± 0.092	0.6469
PCO2 (mmHg)	42.30 ± 3.886	45.22 ± 3.586	45.40 ± 2.821	0.1932
PO2 (mmHg)	295.7 ± 66.22	267.5 ± 41.99	273.0 ± 49.84	> 0.999
BE	− 2.5 ± 2.739	− 0.50 ± 2.881	− 2.000 ± 2.828	0.6697
HCO^3−^	23.35 ± 1.942	21.88 ± 2.606	21.93 ± 1.898	0.4887
TCO^2^	24.50 ± 1.871	23.83 ± 3.43	24.67 ± 3.559	0.9072
SO^2^%	100.0 ± 0.000	99.67 ± 0.516	99.83 ± 0.408	0.7613
Na^+^	137.2 ± 3.545	140.8 ± 1.472	140.8 ± 1.835	0.7634
K^+^	4.867 ± 0.680	4.85 ± 0.217	4.917 ± 0.279	0.5783
Ca^2+^	1.362 ± 0.111	1.172 ± 0.061	1.142 ± 0.085	0.7901
Hct (%)	35.33 ± 9.564	31.67 ± 1.366	32.50 ± 1.643	0.963
Hb (g/dL)	12.02 ± 3.266	10.27 ± 0.554	10.62 ± 0.3312	0.573

P value stands for ECMO versus ECMO + DEX

*Hct* hematocrit, *Hb* hemoglobin

### Kidney injury and functionality

**Fig. 3:**
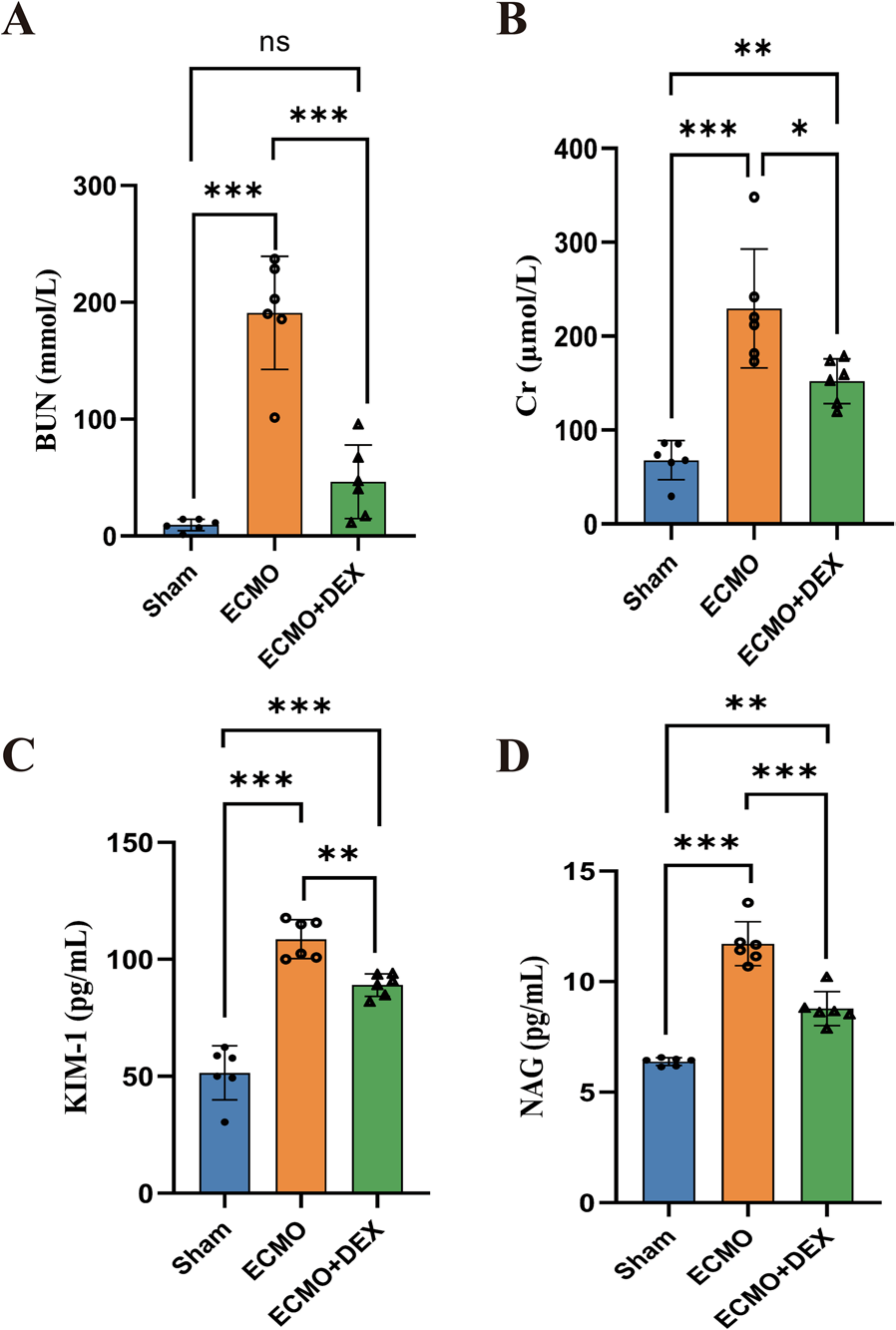
The levels of BUN and Cr in the serum of each group detected by biochemical kit (**A**, **B**). The levels of kidney injury biomarker KIM-1 and NAG (**C**, **D**) in the serum of each group detected by ELISA, *P < 0.05, **P < 0.01, ***P < 0.001

The serum levels of blood urea nitrogen (BUN) and creatinine (Cr) were significantly increased after ECMO. However, we found that DEX significantly reduced the serum levels of BUN and Cr in the ECMO + DEX group compared with that in the ECMO group (Fig. 3A and B). We found that both KIM-1 and NAG were significantly increased in the ECMO group compared with the Sham group. However, these biomarkers of kidney injury were significantly decreased in the ECMO + DEX group compared with the ECMO group (Fig. 3C and D).

### Histopathology

**Fig. 4:**
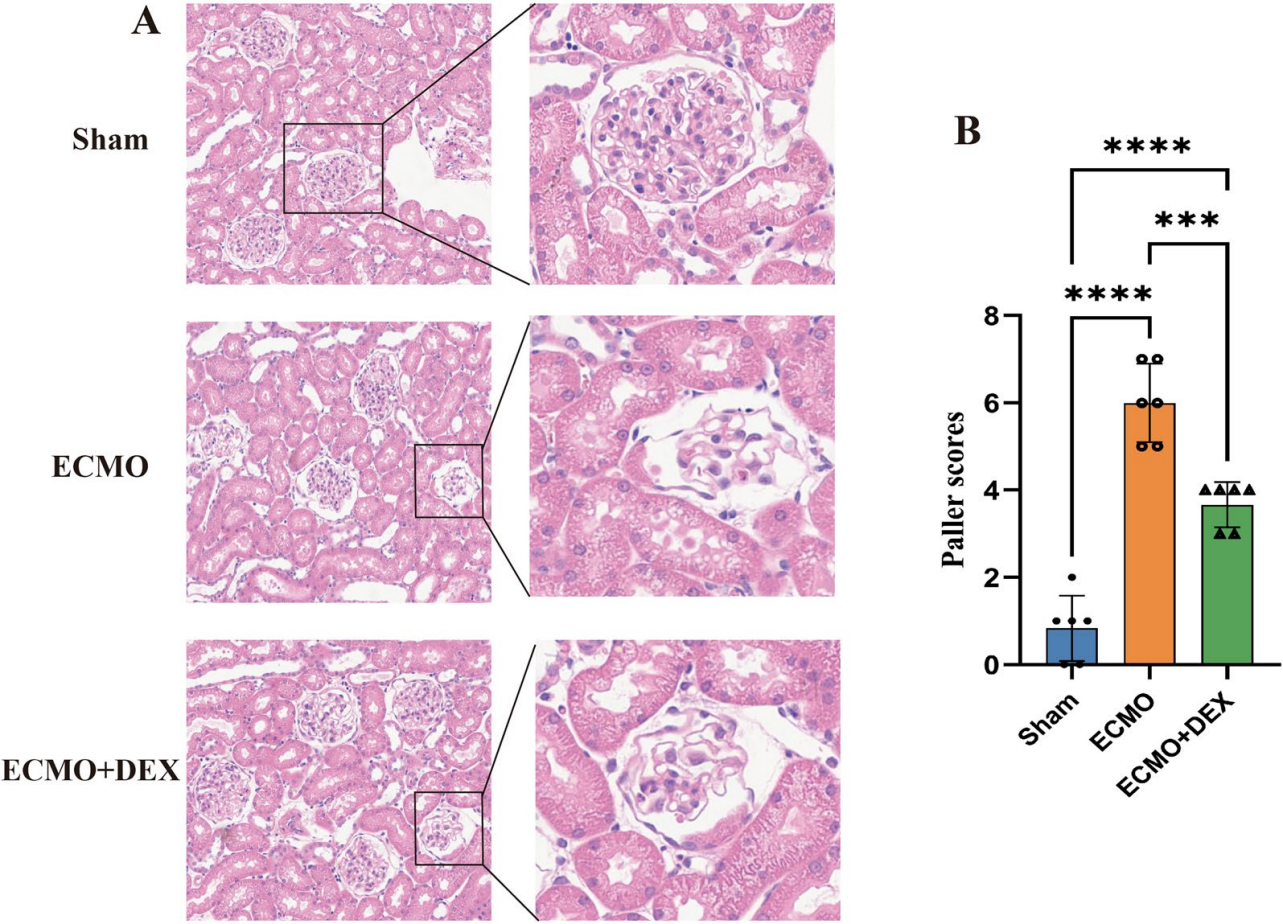
Representative images of kidney tissues with hematoxylin–eosin staining (**A**). The Pallor score of each group was graded from 0 to 8 (**B**), *P < 0.05, **P < 0.01, ***P < 0.001

Hematoxylin eosin staining revealed the extent of pathological changes in the kidney tissue sections, including changes in tubular epithelial smoothness, loss of brush border, cytoplasmic vacuolization, tubular lumen obstruction, and cell necrosis. As shown in Fig. 4A, the pathological analysis showed that the extent of kidney injury increased in the ECMO group and reduced in the ECMO + DEX group, compared with the Sham group. Paller score quantification analysis showed that ECMO caused kidney injury, thereby increasing the Paller score, while DEX significantly reduced kidney injury (Fig. 4B).

### Neutrophil infiltration and inflammatory factors

**Fig. 5:**
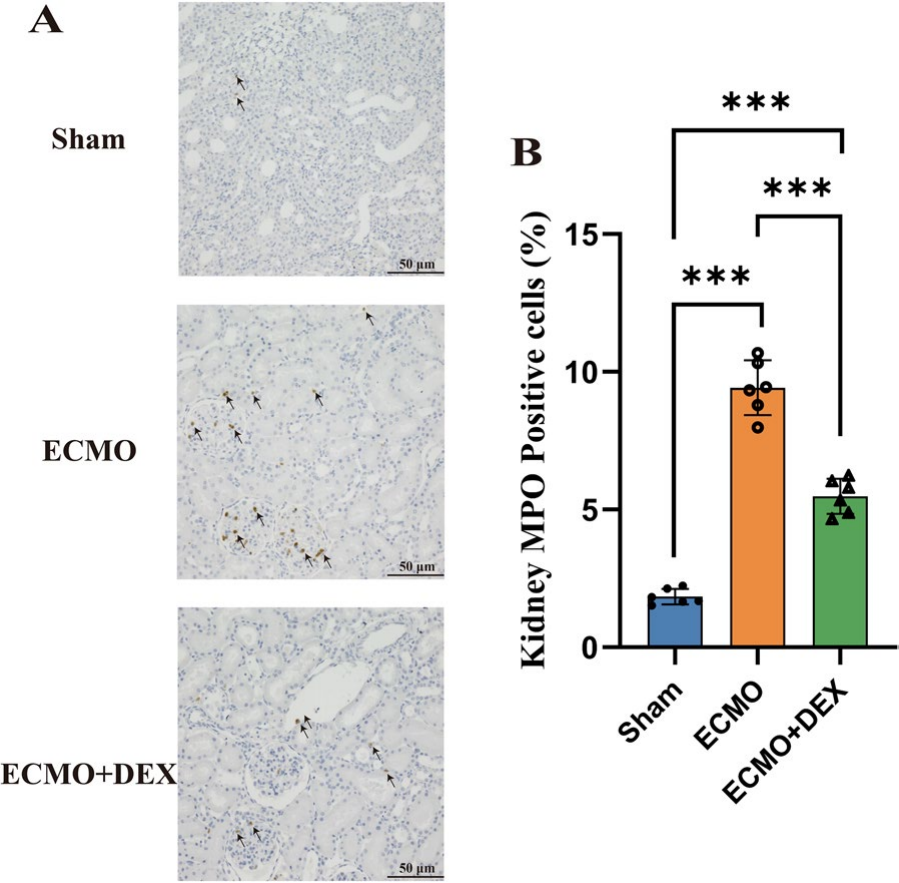
Representative images of kidney tissues with MPO immunohistochemical staining, MPO-positive cells were marked by black arrows (**A**). Three randomly selected fields were used to analyze the activity of myeloperoxidase (**B**), *P < 0.05, **P < 0.01, ***P < 0.001

**Fig. 6:**
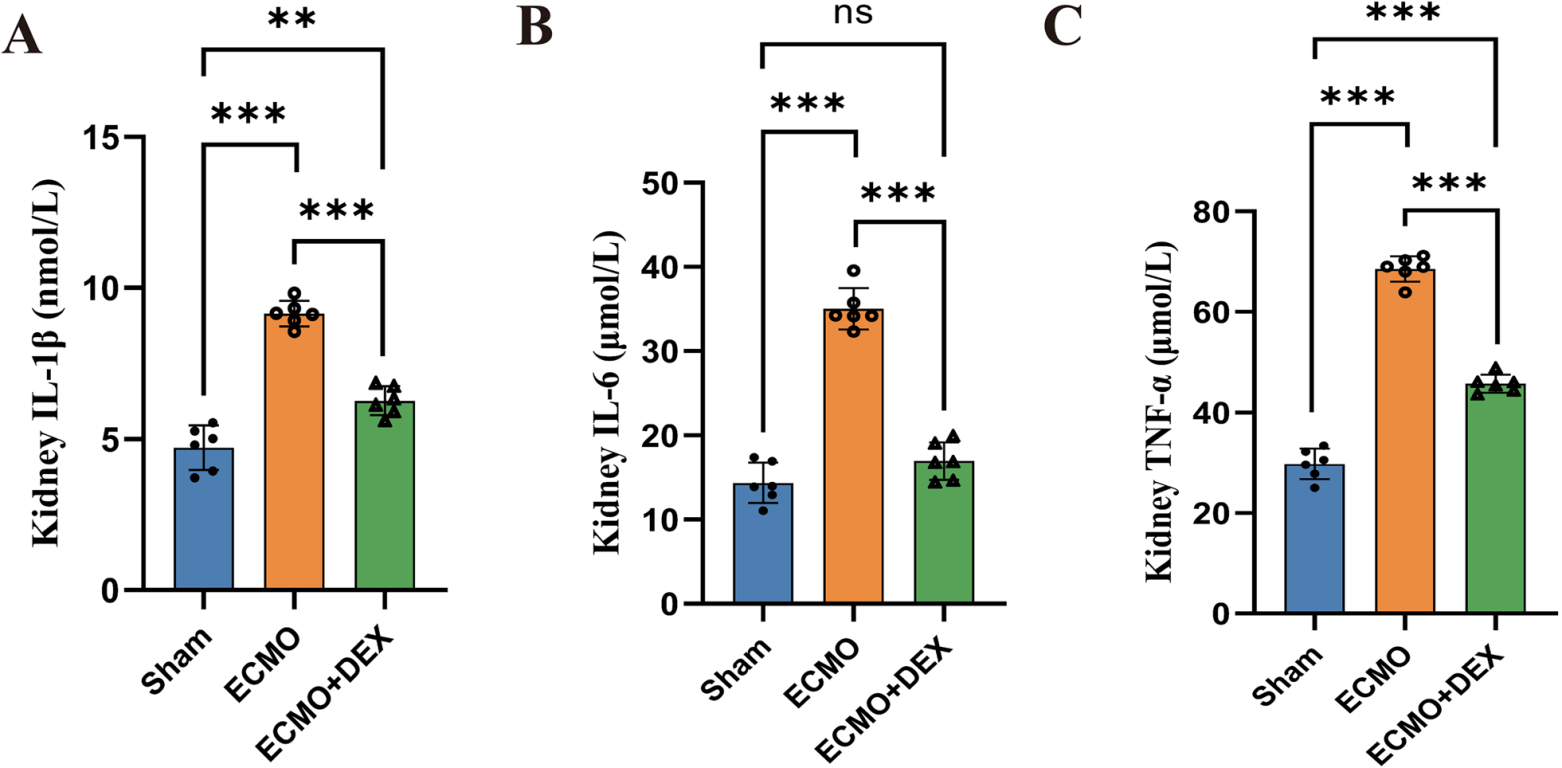
The levels of IL-6, IL-1β, and TNF-α in the serum detected using ELISA Kit (A-C), *P < 0.05, **P < 0.01, ***P < 0.001

Immunohistochemical staining of the kidney tissues of the rats in the Sham, ECMO, and ECMO + DEX groups, using myeloperoxidase antibody, is shown in Fig. 5A and B. We found that MPO + neutrophil infiltrations were greater in the ECMO group than in the ECMO + DEX group. Moreover, the levels of IL-6, IL-1β, and TNF-α in the serum were also significantly increased in the ECMO group. However, we found that these interleukin levels were significantly reduced in the ECMO + DEX group compared with the ECMO group (Fig. 6A–C).

## Discussion

ECMO-induced AKI is a serious complication of ECMO that significantly increases the mortality rates of critically ill patients. Therefore, we designed a study that aimed to investigate the protective effect of a commonly used sedative and analgesic, DEX, on the kidney during ECMO support. We found that DEX alleviates AKI and improves kidney function in a rat model on ECMO support.

ECMO therapy, a temporary respiratory and circulatory support that is widely used worldwide for critically ill patients, has served as a golden option for life support in patients with severe organ failures, especially refractory heart and/or respiratory failure, since the 1970s [1]. However, researchers have found that ECMO is associated with a risk of aggravation and mortality in patients with AKI. Among patients on ECMO, the incidence of AKI ranges from 30 to 78%, while among patients not on ECMO, the incidence is only 20% [15]. Moreover, the incidence of AKI is significantly higher in the VA‐ECMO group compared to those in the VV‐ECMO [15]. Furthermore, 47% to 60% of patients on ECMO require renal replacement therapy (RRT) [16], which is an independent risk factor for hospital death and 90‐day mortality [17]. Additionally, survivors who receive ECMO have a lower risk of AKI and RRT compared with non-survivors [18]. Therefore, AKI might be an independent predictor of poor prognosis for patients on ECMO support.

In this study, we found that kidney injury increased significantly after four hours of VA-ECMO therapy. HE staining of the kidney tissues showed that the Paller score increased significantly during ECMO. Moreover, we also observed an increase in the serum levels of kidney injury biomarkers, including KIM-1 and NAG, and a significant increase in the levels of BUN and Cr, indicating that kidney functionality deteriorates post-ECMO therapy.

The pathophysiology underlying the development of AKI during ECMO support is complicated and multifactorial, and these pathophysiological events can occur at any stage during ECMO therapy. Some of the underlying factors include the management of patients before ECMO therapy, reperfusion after ischemia, worsening of existing systemic disease, release of pro‐inflammatory mediators, breakdown of red blood cells, alteration of the micro and microvasculature of the kidneys, administration of nephrotoxic drugs, abnormal coagulation, and oxidative stress [19].

The used dosage of dexmedetomidine was 50 µg/kg, which was based on other animal studies investigating the protective effect of DEX. In a study by Tao et al. DEX was administered intraperitoneally at 50 µg/kg twice, including 30 min before ischemic reperfusion (I/R) and just the clamp was released. They found DEX attenuates ferroptosis-mediated renal I/R injury and inflammation [20]. In another study by Yang et al., even a higher dosage of 100 µg/kg DEX was given to rats via intraperitoneal, and they found the anti-inflammatory effects of DEX could reduce intestinal I/R injury [21]. The dosage of DEX is usually a lot in organ protection studies. In our study, the entire study process is over four hours, and the elimination half-life of dexmedetomidine is approximately 2 h, in order to avoid secondary administration, and reduce blood dilution we administered a single bolus of dexmedetomidine instead of a continuous application by a syringe pump. Renal perfusion pressure is closely related to renal function and kidney injury. Although the dosage of 50 µg/kg is really high, DEX is a highly selective α2-adrenoreceptor agonist, it has inhibitory effects on the sympathetic nerve system through central α2 agonism, only extremely high dosage (over 1000 µg/kg) have peripherally α1 agonism effect that could cause contraction of peripheral blood vessels, which might have some impact on the hemodynamic parameters. We did not find any significant difference in the mean arterial pressure (MAP) and heart rate between ECMO and ECMO + DEX group in our study. Research by Tanaka et al. found that for patients on VA-ECMO, a higher MAP had a lower incidence of kidney injury [22]. From our point of view, the MAP should be higher than 90 mmHg to provide enough renal perfusion and lower the risk of AKI during VA-ECMO support. The priming fluid used in our study consisted of Ringer’s solution (2.5 ml) and 6% HES130/0.4 (2.5 ml). As is known, hydroxy-ethyl-starch could induce AKI in critically ill patients. The hydroxy-ethyl-starch in the priming fluid could cause AKI in the VA-ECMO group. It is hard to know whether the VA-ECMO caused AKI or the priming fluid did, which is necessary for ECMO. In order to avoid this confounding factor, the same priming fluid was given to the Sham group as well. However, this approach could have led to a volume overload in the sham group. The collection of blood from the rats and the replacement with priming fluid according to the dilutional factor could be an ideal procedure to optimize the experiment.

During ECMO support, injury to the endothelial cells and the extracorporeal surface results in the activation of IL-1β, IL-6, and TNF-α [23]. Consistent with previous studies, we found that the serum levels of IL-1β, IL-6, and TNF-α increased significantly post-ECMO support, which led to the malfunctioning of the microcirculatory system, resulting in a hyperactive immune response and eventually progressing toward AKI. To enhance the tolerance to mechanical ventilation, improve patient–ventilator synchrony, and reduce discomfort, sedation is necessary during ECMO support [24]. Since inadequate sedation can increase the risk of accidental extubation and hemodynamic instability [25], various sedatives and analgesics, including propofol and benzodiazepines, have been used for sedation. However, these sedatives have certain issues that cannot be ignored. For example, the long-term use of propofol is associated with the development of propofol syndrome [26], and benzodiazepines often induce respiratory depression. DEX, on the other hand, results in the pre-synaptic activation of alpha 2-adrenoreceptors, contributing to the inhibition of epinephrine, norepinephrine, and pain signals. It also causes the post-synaptic activation of alpha 2-adrenoreceptors, resulting in a decrease in blood pressure and heart rate. In a variety of settings, it produces a reliable sedative and analgesic effect and has a lower risk of respiratory depression. Moreover, unlike conventional agents, such as etomidate, propofol, and benzodiazepines, it is a non-gabamimetic that can prevent immunologic dysfunction, particularly in patients at risk of infectious complications in the intensive care unit setting [27–29]. Therefore, owing to its analgesic, hemodynamic, sedative, and sympatholytic qualities, DEX has a wide range of clinical applications, making it particularly useful for patients on ECMO support. In our study, we found that the administration of DEX significantly reduced kidney injury during VA-ECMO therapy.

Significantly increased levels of biochemical indicators, such as BUN, Cr, and KIM-1, have the potential to reflect renal dysfunction. KIM-1 and NAG are two biomarkers that are used to detect and assess kidney injury and damage [30]. KIM-1 reportedly regulates renal function recovery and tubular degeneration, and thus, serves as an indicator of tubular injury [31]. In our study, we found that the levels of BUN, Cr, KIM-1, and NAG were significantly decreased in the ECMO + DEX group compared with the ECMO group. Previous studies have shown that DEX can inhibit pro-inflammatory cytokine production, in addition to producing sedative and analgesic effects. A meta-analysis by Wang et al*.* reported a significant decrease in the concentrations of IL-6, TNF-α, CRP, IL-1β, and IL-8, and an increase in IL-10 concentration in surgical patients after DEX administration [32], thereby indicating that DEX possesses anti-inflammatory properties. Consistent with the findings of the above study, our study also found that the levels of IL-6, IL-1β, and TNF-α decreased significantly when DEX was administered before ECMO therapy. Since inflammatory responses result in severe renal dysfunction, therapies involving the inhibition of inflammatory responses as the starting point for treatment may serve as an effective strategy for reducing AKI [33]. Moreover, cell death and inflammation are the major pathophysiology processes underlying AKI [34]. In particular, ferroptosis, necroptosis, and mitochondrial permeability transition-mediated regulated necrosis of tubular cells cause the release of damage-associated molecular patterns, which lead to the recruitment of inflammatory cells, resulting in further injury [35]. In the early stage of AKI, numerous neutrophils are detected in the kidney tissue [36], and renal function is improved following the depletion of neutrophils, thereby indicating the significant role of neutrophils in AKI [37]. Moreover, during the early phase of injury, neutrophils infiltrate the kidney [38] and contribute to organ damage. Although the underlying mechanism is unclear, in this study, we observed that the MPO + neutrophil infiltrations were significantly reduced following DEX administration.

## Limitation

This study has some limitations. Firstly, VA-ECMO lasted only four hours in our study to alleviate damage to red blood cells from the pump. Secondly, we performed VA-ECMO in healthy rats, which is different from clinical practice. Thirdly, this is an animal study with a relatively small sample size, so the conclusion needs further studies to confirm. Fourthly, we administered a single bolus of dexmedetomidine to minimize hemodilution, instead of a continuous application by a syringe pump, which is commonly used in critically ill patients, it remains unknown which way is better to reduce kidney injury. Finally, we used unphysiological high-dosage dexmedetomidine based on literature report, and it is still unclear whether normal dosage in clinical could reduce kidney injury, it is hard to translate this animal model study to humans.

## Conclusion

In conclusion, we found that in a rat model with ECMO the administration of DEX can reduce inflammatory responses in the kidney, thereby alleviating VA-ECMO-induced AKI. We believe that these findings will aid in designing clinical trials that investigate the protective effects of DEX on the kidney during ECMO support, which will indirectly aid in reducing the incidence of AKI and improving patient outcomes.

## Data Availability

The datasets used and/or analyzed during the current study are available from the corresponding author on reasonable request.

## Abbreviations

DEX: Dexmedetomidine
ECMO: Extracorporeal membrane oxygenation
AKI: Acute kidney injury
SD: Sprague–Dawley
BUN: Blood urea nitrogen
Cr: Creatinine
KIM-1: Kidney injury molecule-1
NAG: N-Acetyl-β-D-glucosaminidase
IL-6: Interleukin‐6
IL-1β: Interleukin‐1β
TNF-α: Tumor necrotic factor‐α
ELSO: Extracorporeal life support organization
PEEP: Positive end-expiratory pressure
HE: Hematoxylin–eosin
ELISA: Enzyme-linked immunosorbent assay
RRT: Renal replacement treatment
